# Clinical characteristics of current COVID-19 rehabilitation outpatients in China

**DOI:** 10.1515/med-2023-0771

**Published:** 2023-08-30

**Authors:** Bingxu Chen, Jie Shi, Jie Chen, Yuangang Qiu

**Affiliations:** Department of cardiology, The First Affiliated Hospital of Zhejiang Chinese Medical University (Zhejiang Provincial Hospital of Chinese Medicine), Youdian Road No. 54, Hangzhou, Zhejiang Province, China; Key Laboratory of Integrative Chinese and Western Medicine for the Diagnosis and Treatment of Circulatory Diseases of Zhejiang Province, Hangzhou, Zhejiang Province, China; The First Affiliated Hospital of Zhejiang Chinese Medical University, (Zhejiang Provincial Hospital of Chinese Medicine), Hangzhou, Zhejiang Province, China

**Keywords:** COVID-19, COVID-19 rehabilitation clinic, pneumonia, elderly patients, chinese medicine

## Abstract

To understand the clinical characteristics of omicron in COVID-19 Rehabilitation Clinic after the current shift of dynamic zeroing policy, we consecutively collected the patients’ data who visited in COVID-19 Rehabilitation Clinic of a Grade-A tertiary hospital in Hangzhou, Zhejiang Province, from January 3 to January 10, 2023, analyzed related data and then compared the pneumonia between elderly and non-elderly groups. The results showed that 95.68% of the patients in COVID-19 Rehabilitation Clinic had symptoms, 70.10% had a dry cough, 12.36% had abnormal complete blood count or C-reactive protein, 19.35% had electrolyte disorder, and 2% had abnormal troponin or creatine kinase-MB. 40.45% of patients had abnormal lung CT findings, among them 86.49% of elderly patients had abnormal lung CT findings, and the utilization rate of glucocorticoids in COVID-19 Rehabilitation Clinic was only 5.98%, although people are all susceptible to getting the COVID-19 infection, the elderly are more prone to getting pneumonia, and the glucocorticoids utilization rate is relatively insufficient. It is needed to be stressed that Chinese medical staff should pay more attention to the elderly patients who are vulnerable to getting pneumonia during this period.

## 1 Introduction

COVID-19 is a worldwide epidemic, currently in the omicron variant. For Western countries, the virulence and pathogenicity of omicron are greatly reduced. At present, many countries have adopted mRNA vaccination, which has mostly achieved the effect of herd immunity.

Since 2019, China has adopted the policy of dynamic-zeroing policy against COVID-19 virus strains (alpha, beta, and delta) and achieved effective clinical results. Nevertheless, since the spread of the omicron variant from South Africa, most countries in the world have gradually adopted herd immunity measures against the more transmissible (*R*0 = 18), less virulent virus [1]. In a recent view of the characteristics of Omicron, the Chinese government ended the dynamic-zeroing policy since December 8, 2022, and shifted to the policy of rapid peak crossing and herd immunity. By mid-January, more than 80% of the public in some parts of China had been infected with Omicron [2] or were being infected with Omicron.

Scientists have reported that omicron also has the ability to cause syncytia formation, which is often seen in the lungs of patients dying of COVID-19 [3]. Vogel et al. found that omicron tends to evade the bronchi tissue compared to the delta variant, but it also causes the lung tissue damage [4]. Although it is unclear about elderly patients with omicron infection in China, we thought that the elderly are more susceptible to the infection and might need more attention during this period. For patients with pneumonia, some scholars advised to use short-term corticosteroid to prevent the interstitial lung disease [5]. However, the impact of omicron on Chinese people and the cognition and treatment of Omicron in Chinese medical staff on the disease is still unclear. Therefore, this study was based on real-world data review and analysis of patients who visited the COVID-19 Rehabilitation Clinic to provide related data for future treatment.

## 2 Methods

### 2.1 Study patients and location

From January 3 to January 10, 2023, patients who visited COVID-19 virus rehabilitation at Zhejiang Hospital of Traditional Chinese Medicine (TCM) clinic for the treatment of COVID-19 infection were consecutively enrolled in this study. This study location is mainly at Zhejiang Hospital of TCM which has combined TCM and modern medicine and has acquired solid trust from the surrounding neighborhood. Exclusion criteria included patients who disagreed with the study, patients who cannot apprehend the consent form, and patients with cognition problems.

### 2.2 Study design and procedures

All patients received COVID-19 Rehabilitation Clinic doctors’ diagnosis and treatment without any intervention by the investigators. After the treatment, we would get consent from patients and record their diagnosis and treatment plan from the outpatient clinical computer system. And then we check the basic information including sex, age, and medical history, collect the CT scan, blood test, lab results, and related treatment plan, and put clinical data into the relevant database.

### 2.3 Statistical analysis

Statistics were performed using the SPSS 1200 for Windows software (SPSS Institute, Chicago, IL, USA). Qualitative data with normal distribution were presented as mean ± standard deviation and compared using the Student’s *t*-test. Skewed data were presented as median (min–max) and compared using the Mann–Whitney *U* test. Quantitative data were presented as frequencies and compared by the *χ* ^2^ or Fisher’s exact test. A *P*-value of <0.05 was considered statistically significant.

A total of 602 patients from January 3 to January 10, 2023, in the COVID-19na-virus Rehabilitation Clinic of a Grade-A tertiary hospital in Hangzhou, Zhejiang Province, was investigated. The basic information, chief complaint, course of disease, past medical history, test results, and related treatment were retrospectively recorded and analyzed. SPSS 20 was used for statistical analysis.


**Informed consent:** Informed consent has been obtained from all individuals included in this study.

## 3 Results

There were 602 patients in total, including 220 males and 382 females, with an average age of 43.8 ± 16.6, 508 young and middle-aged patients (84.39%), and 94 elderly patients (15.61%). The average course of disease was 9.71 ± 5.88 days. Among them, 26 were asymptomatic (4.32%), 5 had night sweats (0.83%), 4 had nausea and vomiting (0.66%), 1 had tinnitus (0.16%), 25 had fatigue (4.15%), 25 had fever (4.15%), 6 had diarrhea (0.99%), 422 had a cough (70.10%), 6 had phlegm ( 0.99%), 3 patients had phlegm mixed with phlegm (0.5%), 7 patients had muscle pain (11.63%), 2 patients had bitterness in mouth (0.33%), 10 patients had dizziness (16.61%), 12 patients had palpitation (1.99%), 53 patients had chest tightness (8.80%), 13 patients had chest pain (2.16%), 3 patients had anosmia (0.5%), 3 patients had throat discomfort (0.5%), and 1 patient had syncope (0.16%). See Table 1 for details.

**Table 1:** Distribution of chief complaints of COVID-19 rehabilitation outpatients

Chief complaints	Total (*n*)	%
Tinnitus	1	0.17
Syncope	1	0.17
Bitter taste in mouth	2	0.33
Hemoptysis	3	0.5
Anosmia	3	0.5
Sore throat	3	0.5
Nausea and vomiting	4	0.66
Night sweat	5	0.83
Diarrhea	6	0.99
Expectoration	6	0.99
Muscle soreness	7	1.16
Dizzy	10	1.66
palpitation	12	1.99
Chest pain	13	2.16
Weakness	25	4.15
Asymptomatic	26	4.32
Chest tightness	53	8.80
Dry cough	422	70.10

Twenty-three patients (3.82%) had other diseases, 10 patients (1.67%) had hypertension, 2 patients (0.33%) had diabetes, 1 (0.16%) had anemia, 1 (0.16%) had coronary heart disease, 1 (0.16%) had Chronic obstructive pulmonary disease (COPD), 2 patients (0.33%) had osteoporosis, 1 (0.16%) had stroke history, and 1 (0.16%) had asthma. Two patients (0.33%) had well-controlled tumor history and 1 (0.16%) had chronic kidney disease (CKD). See Table 2 for details.

**Table 2:** Distribution of basic diseases of patients in COVID-19 rehabilitation clinic

Past history	Total (*n*)	%
Anemia	1	0.16
Coronary artery disease	1	0.16
COPD	1	0.16
Stoke	1	0.16
Asthma	1	0.16
CKD	1	0.16
Diabetes	2	0.33
Osteoporosis	2	0.33
Tumor	2	0.33
Hypertension	10	1.67

A total of 178 patients took the complete blood cell count (CBC), 16 (8.99%) had abnormal leukocyte, 14 (7.87%) had abnormal neutrophils, 20 (11.24%) had abnormal lymphocytes, 13 (7.3%) had abnormal hemoglobin, and 20 (11.24%) had abnormal platelets. Twenty-two patients (12.36%) had abnormal C-reactive protein (CRP), 31 patients take electrolyte analysis examination, including 1 patient with high potassium (3.23%), 1 patient with low potassium (3.23%), 6 patients with low sodium (19.35%), 1 patient with high sodium (3.23%), 1 patient with high chlorine (3.23%), 1 patient with low chlorine (3.23%), and 1 patient with low calcium (3.23%). One hundred patients took the creatine kinase-MB (CK-MB) and troponin (TNI), 2 patients (2%) had abnormal TNI and 1 patient (1%) had abnormal CK-MB. See Table 3 for details.

**Table 3:** laboratory test of COVID-19 rehabilitation outpatients

	Total (*n*)	%
Complete blood cell test	178	29.57
	16	8.99
Increase in neutrophil proportion	14	7.87
Decrease in lymphocytes	20	11.24
Decrease in hemoglobins	13	7.3
Increase in platelets	20	11.24
Increase in CRP	22	12.36
Electrolytes test	31	5.15
Hyperkalemia	1	3.23
Hypopotassium	1	3.23
Hypernatremia	1	3.23
Hyponatremia	6	19.35
Hyperchlorine	1	3.23
Hypochlorine	1	3.23
Hypocalcemia	1	3.23
Myocardial enzymes	100	16.61
Increase in TNI	2	0.02
Increase in CK-M	1	0.01

A total of 440 patients took lung CT examination, 36 (8.18%) asked for CT examination voluntarily. 72.05% of young- and middle-aged patients took CT examination and 78.72% of elderly patients took CT examination. 40.45% of patients were found abnormal lung CT. See Table 4 for details.

**Table 4:** Pulmonary computer tomography (CT) results of COVID-19 Rehabilitation Clinic

Results	Non-elderly patients (366)	Elderly patients (74)	Chi-square	*P* value
Normal	252 (68.85)	10 (13.51)	78.25	<0.001
Abnormal	114 (31.15)	64 (86.49)		
Little (＜10%)	75 (20.49)	18 (24.32)		
Mild (10–30%)	24 (6.56)	28 (37.84)		
Multiple (30–50%)	15 (4.10)	17 (22.97)		
White lung (＞50%)	0	1 (1.35)		

Note: 2 groups are analyzed by Chi-square test, *P* < 0.05 means significant statistically.

Five (0.83%) patients were admitted to wards for further treatment, 36 (5.98%) patients were treated with glucocorticoids therapy, 23 (3.82%) patients were treated with antibiotics, and 141 (23.42%) patients were treated with TCM, See Table 5 for details.

**Table 5:** Treatment prescriptions for COVID-19 rehabilitation outpatients

Treatment	Total (*n*)	%
Hospitalization	5	0.83
Glucocorticoid	36	5.98
Antibiotic	23	3.82
TCM	141	23.42

## 4 Discussion

Di Mitri et al. [6] collected the COVID-19 patients consecutively in the out-patient clinic and found that 17% of these patients developed pneumonia, and our study found that there was a higher prevalence of patients with pneumonia who were diagnosed with COVID-19 (40.45 vs 17.0%). The underlying reason might be that during the strict quarantine policy, people work out less than before and they are in a depressed mood during the period that might inactivate their immunity potent, and the majority were not exposed to the other relevant pathogens, which led to the general susceptibility of the Chinese population. The sharp shift in policy also caused the huge pressure on medical resources, and many patients failed to seek any medical treatment in time. In addition, most people were injected with inactivated vaccines other than the mRNA vaccine, which has been proven less effective in the health protection.

Our results showed that the COVID-19 Rehabilitation Clinic is mainly targeted at patients who still have symptoms after self-quarantine at home, and most patients have no other chronic diseases in the past. Only 3.82% of the patients had other diseases in the past, mostly hypertension (1.67%). Patients with acute onset and severe situation are mostly hospitalized through the pre-hospital ambulance system, which is not discussed in the article.

70.10% of patients went to the COVID-19 Rehabilitation Clinic with recurrent cough, all of which were post-COVID-19 irritant dry cough. The omicron virus may cause airway hypersensitive, and thus, patients’ exposure to cold air and other stimulus cause local airway spasm, resulting in dry cough. 0.99% of patients had a cough with yellow sputum, which is considered to be related to the apoptosis of epithelial cells and immune cells after COVID-19 infection and may be partly due to the presence of bacterial co-infection. 0.50% of the patients had sputum mixed with blood. After checking patients’ relevant history, it was found that the upper respiratory tract of these patients was relatively dry due to self-isolation at home with higher temperature caused by the heating system, and nearly absence of air humidity. Therefore, the capillary in the upper respiratory tract burst when coughing forcefully.

8.80% of patients came to see the doctor because of chest tightness, which may be related to the impaired ventilation diffusion function caused by pneumonia. 2.16% of patients visited the clinic for recurrent chest pain. Some might be linked with the pleura inflammation. 2% of patients who took the cardiac enzyme test are abnormal, suggesting myocardial damage, which may be related to the direct damage to the myocardium caused by vaccination or COVID-19 [7].

0.5% of patients have anosmia, which may be related to the damage of Bowman gland cells caused by the direct destruction of nerve support cells by COVID-19, mucus protection impairment in the olfactory epithelium, and energy exhaustion supporting the nerve cells [8].

0.66% of patients had nausea and vomiting, which might be related to abnormal secretion of interleukin after infection, and 0.99% of patients had diarrhea, which might be related to direct damage in the gastrointestinal tract by the novel COVID-19 or vagus nerves hyperactivity during rehabilitation [9].

11.63% of patients had musculoskeletal soreness, which may be related to disorder in immune systems, in which immune cell-mediated cytokines or relevant neural transmitters attack connective tissue causing the soreness [10].

0.83% of patients had night sweats, which may be related to vagus nerves hyperactivity during rehabilitation. 4.15% of patients presented with consistent fatigue which may be related to the slow recovery of the body and deficiency in the nutrition provision. 16.61% of patients had dizziness, and no obvious abnormalities were found in head CT which may be related to vestibular neuritis caused by the possible infection of the COVID-19. 0.16% of patients had tinnitus, which may also be related to the vestibular neuritis. 0.16% of patients had syncope during COVID-19 recovery, which may be related to hypoglycemic episodes caused by poor appetite, and further follow-up examination is needed for the patient. 29.57% of patients underwent CBC test, 8.99% of the patients had abnormal white blood cells, 7.87% had abnormal neutrophil proportion, 11.24% had abnormal lymphocyte proportion, 7.3% had abnormal hemoglobin, 11.24% had abnormal platelet proportion, 12.36% had abnormal CRP, and abnormal white blood cells in these patients were all mildly elevated with less than 15 × 10^9^/L, accompanied by an increase in the proportion of neutrophils, a decrease in the proportion of lymphocytes, and an increase in CRP, which indirectly indicated the infection of the COVID-19 virus. 11.24% of patients had abnormal platelet elevation, which may be related to the stimulation of platelet production by inflammatory factors after infection with COVID-19, or the hypercoagulable state in the menstrual period of female patients. Mild anemia occurred in 7.3% of patients, which may be related to underlying diseases, poor intestinal absorption, or insufficient iron intake.

5.15% of the patients underwent electrolyte examination, among whom 3.23% had hyper-potassium, which was related to chronic renal insufficiency, and 3.23% had hypo-potassium, which might be related to long-term use of thiazide drugs. 19.35% of the patients with lots of water intake and light diets in the course of rehabilitation had mild hypo-natremia, partially due to the acceptation of twisted information from public media. 3.23% of patients had low calcium, which was related to their past medical history and lack of outdoor exercise.

73.09% of patients underwent lung CT examination, and 43.18% of patients had pneumonia according to the CT results. Among them, the pneumonia proportion in young- and middle-aged patients was 31.15%, while the pneumonia proportion in elderly patients without underlying diseases was as high as 86.49%, not to mention 1.35% white-out lungs among them. Although the omicron has a less toxicity compared to the alpha/delta, such a high proportion of pneumonia and severe disease indirectly reflects that Omicron’s disease progression in China is much different from that in other countries. On the one hand, it may be because only the symptomatic patients seek out for outpatient treatment, leading to the existence of bias. On the other hand, it is also indirectly suggested that although the toxicity of omicron has waned in comparison with the beginning, it is far from insignificant in the Chinese population. First, some elderly people have not been regularly vaccinated with domestic inactivated vaccines; second, compared with foreign countries, RNA vaccines that have more efficacy to prevent COVID-19 have not been widely applied in the Chinese people; moreover, the preceding dynamic zero elimination policy has resulted in the most Chinese people not being exposed to COVID-19. As a result, Chinese people are generally susceptible to COVID-19. Moreover, the abrupt turn of the policy caused a chaotic situation among Chinese people, for instance, some people cannot obtain any medical resources, and most of them fail to get any antipyretic, decongestants, expectorants, antihistamines, and antitussive and apophlegmatisant drugs at the beginning and some patients with high fever relieve their symptoms just by physical cooling or using primitive non-scientific methods, resulting in dehydration, internal environment disorders, or even ending up into liver or kidney dysfunction, which greatly increased the disease progression and the medical staff burdens. This is the current situation in China’s well-developed city, and the situation in under-developed rural hinterland may be even worse than that. For such reasons, medical staff in those areas require to be more vigilant in the coming months.

In terms of treatment, 0.83% of patients were assessed by clinicians and admitted for further treatment. Although the guidelines recommend that the COVID-19 pneumonia requires glucocorticoids and antiviral drug treatment rather than antibiotic treatment [11,12], merely 5.98% of patients received glucocorticoid therapy and up to 3.82% received antibiotics; the reason for this is that due to the serious reduction of medical staff in the respiratory department and the shortage of health carers, the COVID-19 Rehabilitation Clinic physicians are mostly made up of various auxiliary departments as a last resort, who are from cardiology, acupuncture, rehabilitation, ophthalmology, orthopedic, and other departments. Some doctors failed to grasp the relevant guidelines and recommendations in time. Additionally, they lack experience in the application of glucocorticoids leading to the relevant deficit of treatment. Since there is no antiviral drug in this outpatient department, and most patients have a longer course than 7 days, the outpatient department patients cannot receive any antiviral treatment.

About 23.42% of the patients were treated with the Chinese medicine prescription. The dialectical interpretation of the new coronavirus in TCM is Qi and Yin deficiency, spleen deficiency and loss of transport, and residual illness. Therefore, we have two Chinese prescriptions for two types of COVID-19 patients. One is the COVID-19 treatment formula, which contains forsythia, honeylonea, bitter platycodon, mint, bamboo leaves, Schizonepeta, stir-fried beef nuts, ephedra, bitter almonds, licorice, gypsum, sand ginseng, raw jade bamboo, mulberry leaves, etc., which is used for fever and other related symptoms. Another Chinese medicine treatment formula is for patients who have had COVID-19 infection but still remain some symptoms like cough, sneeze and fatigue. For some patients have generally accepted the Chinese traditional medicine, and this hospital also is a TCM research hospital, so the application rate of Chinese traditional medicine was relatively high. Besides, all patients were treated with anti-symptomatic therapy.

However, the efficacy has not been confirmed, and further follow-up observation is required. Based on the results above, we suggest that medical staff should be more vigilant, and patients with persistent COVID-19 symptoms should be given a CT examination and other tests to exclude related diseases. Also, the understanding of the treatment of COVID-19 should be strengthened and the treatment of COVID-19 should be standardized in medical staff, and physicians should diagnose these patients as soon as possible and prescribe antiviral drugs in time.

For patients with pneumonia, physicians should use hormones in short terms to avoid fibrosis of lung tissue and try not to use antibiotics for COVID-19 patients without bacterial infection. Finally, we suggested that the government should adopt mRNA vaccine to protect the high-risk group in China.

First of all, this article only selected patients from a Grade-A tertiary hospital in a coastal city in eastern China, without fully representation for other areas. Second, due to the special feature of the COVID-19 Rehabilitation Clinic, the clinic generally does not accept patients in emergency or severe situations, so it is impossible to actually estimate the serious patients’ proportion and mortality caused by Omicron infection. Furthermore, we did not follow up on out-patient patients at present, so we cannot predict the short- and long-term omicron outcomes for these patients.
